# Epidemiology and clinical management of nail clipping in dogs under UK primary veterinary care

**DOI:** 10.1111/jsap.70002

**Published:** 2025-08-05

**Authors:** L. A. Ahmed, M. Somarriba, D. C. Brodbelt, D. B. Church, D. G. O’Neill

**Affiliations:** ^1^ Applied Animal Behaviour and Welfare University of Edinburgh Edinburgh UK; ^2^ School of Veterinary Medicine & Bioscience Scotland’s Rural College (SRUC) Edinburgh UK; ^3^ Pathobiology and Population Science The Royal Veterinary College Hatfield UK; ^4^ Pathobiology and Population Science The Royal Veterinary College Hatfield UK

## Abstract

**Objectives:**

This study aimed to report the frequency, risk factors and clinical management of nail clipping in dogs under primary veterinary care across the United Kingdom during 2019 within the VetCompass Programme.

**Materials and Methods:**

From a population of 2,250,741 dogs under veterinary care, 2440 nail clipping cases (3380 events) were randomly selected. Data on demographics, clinical rationale and nail details were extracted from clinical notes. A cross‐sectional analysis was conducted to estimate the 1‐year (2019) proportion of nail clipping and to identify associations with demographic risk factors.

**Results:**

The 1‐year proportion of nail clipping was 5.64% (95% CI 5.43 to 5.86). Breeds with the highest odds for nail clipping compared to non‐designer crossbreds included Chihuahua (OR 2.21, 95% CI 1.88 to 2.60), beagle (OR 2.09, 95% CI 1.54 to 2.83) and Greyhound (OR 2.02, 95% CI 1.37 to 2.97). Dogs aged (1 to 2) years had the highest odds (OR 1.61, 95% CI 1.35 to 1.92). Nail clipping was the primary reason for veterinary visits in 59.4% of events, with overgrown or ingrown nails (12.66%) and broken claws or dewclaws (8.84%) being the most common clinical justifications.

**Clinical Significance:**

The findings highlight the importance of veterinary‐led nail care guidance to canine welfare, with breed‐specific considerations. Further research is needed to better understand how the underlying biological and behavioural factors are affecting the variables identified here and contribute to nail clipping probability. Nail clipping should be prioritised in veterinary education and care strategies to address its clinical and welfare implications effectively due to its high frequency.

## INTRODUCTION

Nail (technically claw) clipping in dogs is a husbandry procedure generally aimed at maintaining an optimal length of the nails by shortening them for better health and function (Orpet & Welsh, 2010). Nail clipping is an important component of good canine husbandry, with two‐thirds of dog owners reporting their dogs’ nails are clipped twice or more frequently annually either under veterinary care, directly by the owners or at the groomers (Edwards et al., 2022). Understanding the frequency and risk factors for nail clipping and the welfare implications from these nail clipping procedures carried out in primary veterinary care settings can provide valuable insights in recognising potential health benefits, such as the prevention of overgrown nails and associated injuries, and also help address potentially associated welfare challenges, such as stress and discomfort to the dogs, during the procedure. This knowledge can contribute to improved guidelines and recommendations for canine nail care, ultimately enhancing the overall well‐being and quality of life for dogs.

Currently, neither clinical guidelines nor scientific literature provides a useful standardised definition for “normal” or “optimal” nail length. In dogs, nails grow continuously and will eventually overgrow if not worn down naturally or clipped (Carbonell Buj & Farrell, 2019). Overgrown nails are reported as the fourth most frequently diagnosed disorder in dogs under primary veterinary care in the United Kingdom (O'Neill et al., 2021). Overgrown nails can cause discomfort and mobility issues (Hughes & Soloman‐Kretay, 2007) and might eventually in‐grow into the pads, causing pain, injury and infection (Miller et al., 2012). Nail growth rate in dogs overall has been estimated at 0.8 to 1.9 mm/week (Jackson & Marsella, 2012) but information on differential growth rates across digits or breeds was not provided in that study. Despite reported high frequency of nail clipping in dogs and the links to health issues (Olsson Wiberg, 2024), there is remarkably little evidence reported on how factors such as breed, bodyweight, age or sex may affect the odds of nail clipping. Overgrown nails was identified as one of the most underfunded conditions for research in dogs (Skipper et al., 2024). Identifying risk factors for nail clipping can guide targeted interventions and personalised recommendations for optimal nail care.

Owners report often using various combinations of external services and also themselves for clipping their dog's nails (Edwards et al., 2022), but evidence on the relative uptake of these different services for nail clipping or how different nail clipping procedures may affect the welfare of these dogs is minimal. However, given that 5.52% of dogs under primary veterinary care have been reported with overgrown nails annually (O'Neill et al., 2021) and that torn or broken nails were reported in the top 10 most common conditions reported by owners in the United States, affecting 5.66% of purebreds and 4.83% of crossbreds (Forsyth et al., 2023), it is clear that many owners rely heavily on veterinary primary care to deal with overgrown nail problems. Analysis of anonymised primary care veterinary records should therefore provide valuable insights from the veterinary perspective into risk factors for nail clipping and enable exploration of possible welfare implications that bridge the gap between routine clinical care and academic research.

Using anonymised veterinary clinical data from the VetCompass Programme (VetCompass, 2025), this study aimed to report on proportional nail clipping and risk factors for nail clipping in dogs under primary care veterinary clinics in the United Kingdom during 2019. The study also aimed to report on the clinical management of nail clipping procedures in dogs. The study placed a particular focus on breed as a risk factor for nail clipping. These results could assist veterinary practitioners, welfare scientists, breeders and owners with a stronger evidence base to understand the frequency and associated welfare benefits/harms of nail clipping in dogs.

## MATERIALS AND METHODS

The study population included all dogs under primary veterinary care at clinics participating in the VetCompass Programme during 2019. Dogs under veterinary care were defined as those with ≥1 electronic health record (EHR) (free‐text clinical note, treatment or bodyweight) recorded during 2019. VetCompass collates de‐identified EHR data from primary care veterinary practices in the United Kingdom for epidemiological research (VetCompass, 2025). Data fields available for each animal included fixed values for species, breed, date of birth, sex and neuter status, along with date‐specific information on free‐form text clinical notes, bodyweight and treatment.

A cohort study design with a cross‐sectional analysis was used to estimate the 1‐year (2019) proportion of nail clipping and to explore associations with demographic risk factors for nail clipping in this population. Based on prior evidence for 5.52% prevalence of overgrown nails under primary veterinary care in the United Kingdom (O'Neill et al., 2021), power calculation estimated that a study sample of 31,605 dogs was needed to estimate proportional risk for a nail clipping event occurring in 5.52% of dogs with a 0.25% acceptable margin of error at a 95% confidence level from a national UK population of 8 million dogs (Asher et al., 2011; Dean et al., 2022). Ethical approval was given by the RVC Social Science Research Ethical Review Board (SSRERB) (reference number SR2018‐1652).

The case definition for a nail clipping case required evidence in the clinical records indicating that the dog had at least one nail shortened at any date from January 1, 2019 to December 31, 2019. Case finding involved initial screening of all 2,250,741 study dogs for candidate nail clipping cases by searching the clinical free‐text field using the search terms (nail*, claw*, clip*, trim*, dewclaw* and pedicure*). Candidate dog IDs from these searches were merged, and the clinical notes of a random sample of candidates were manually reviewed to evaluate against the case definition. For each confirmed nail clipping case, additional information was manually extracted on each episode of veterinary care related to nail clipping during 2019. Some dogs had data extracted on multiple nail clipping events. Data extracted for each nail clipping event included the clinical and motivational rationales recorded for the nail clipping, which nails were clipped, and the reason for clipping the nails. Risk factor analysis included a random sample of 795,891 dogs that had not been screened as candidates as the non‐cases.

Breed descriptive information entered by the participating practices was cleaned and mapped to a “VetCompass breed list” derived and extended from VeNom Coding, which included recognised purebred breeds and designer breed terms (The VeNom Coding Group, 2024). A *breed purity* variable categorised all dogs of recognisable breeds as “purebred,” dogs with contrived names generated from two or more purebred breed terms as “designer crossbreed” crossbreds (purposely bred crossbreeds) and dogs recorded as mixes of breeds but without a contrived name as “crossbred” (The Kennel Club, 2025). A breed variable included any breed with >30 dogs manually checked to meet the nail clipping case definition or any breed with >10 confirmed nail clipping cases, with all remaining dogs grouped as “other.” Based on breed information, a skull shape variable categorised dogs as dolichocephalic, mesocephalic, brachycephalic, unrecorded (O'Neill, Packer, et al., 2020). A coat length variable categorised breeds by hair‐coat length (short, medium, long, hairless and uncategorised). A chondystrophy variable categorised breeds as chondystrophic, non‐chondystrophic and unrecorded. A *Kennel Club breed group* variable classified breeds recognised by the UK Kennel Club into their relevant breed groups (Gundog, Hound, Pastoral, Terrier, Toy, Utility and Working) and all remaining types were classified as non‐Kennel Club recognised (The Kennel Club, 2025). Consistent with previously used methods (O'Neill et al., 2021), neuter status was defined by the final available EHR value. Adult bodyweight was defined as the median of all bodyweight (kg) values recorded for each dog after reaching 18 months old and was categorised as follows: <10.0, 10.0 to <15.0, 15.0 to <20.0, 20.0 to <25.0, 25.0 to <30.0, 30.0 to <40.0, 40.0 to <50.0, 50.0 to <60.0 and ≥60.0. Age (years) was defined for each dog on December 31, 2019 and was categorised in 1‐year bands to 24 years.

Following internal validity checking and data cleaning in Excel (Microsoft Office Excel 2013, Microsoft Corp.), analyses were conducted using IBM SPSS Statistics Version 27.0.1 (IBM SPSS, 2024). Continuous variables were checked for normality. The mean [standard deviation (SD)] was reported for normally distributed data and median [interquartile range (IQR), range] reported for non‐normally distributed data. Annual proportion (prevalence) with 95% confidence intervals (CI) described the probability of at least one nail clipping event at any point during 2019. Because the sampling design involved verification of a subset of the candidate cases, the annual proportion was calculated as the count of confirmed cases (overall or by breed) divided by the notional study population overall or for that breed. The notional study population was calculated as the true analysis population multiplied by the proportion of the candidate cases that were manually checked overall or for that breed. The CI estimates were derived from standard errors, based on approximation to the binomial distribution (Kirkwood & Sterne, 2003).

Risk factor analysis used binary logistic regression modelling to evaluate univariable associations between risk factors (*breed*, *skull shape*, *haircoat length*, *chondystrophy*, *breed purity*, *Kennel Club recognised breed*, *Kennel Club breed group*, *adult bodyweight*, *age*, *sex and neuter*) and being a nail clipping case during 2019. Because breed was a factor of primary interest for the study, variables derived from the breed information and therefore highly correlated with breed (*haircoat, skull shape, chondystrophy, breed purity, Kennel Club‐recognised breed and breed group*) were excluded from initial breed multivariable modelling. Instead, each of these variables individually replaced the *breed* variable in the final breed‐focused model. *Adult bodyweight* (a defining characteristic of individual breeds) also replaced breed in the final breed‐focused model. Risk factors with liberal associations in univariable modelling (P < 0.2) were taken forward for multivariable evaluation. Multivariable model building used the SPSS automated backwards stepwise elimination. The Hosmer‐Lemeshow test was used to evaluate the quality of the final model fit (Hosmer Jr et al., 2013). Statistical significance was set at P < 0.05.

## RESULTS

### Annual proportional nail clipping

**FIG 1:**
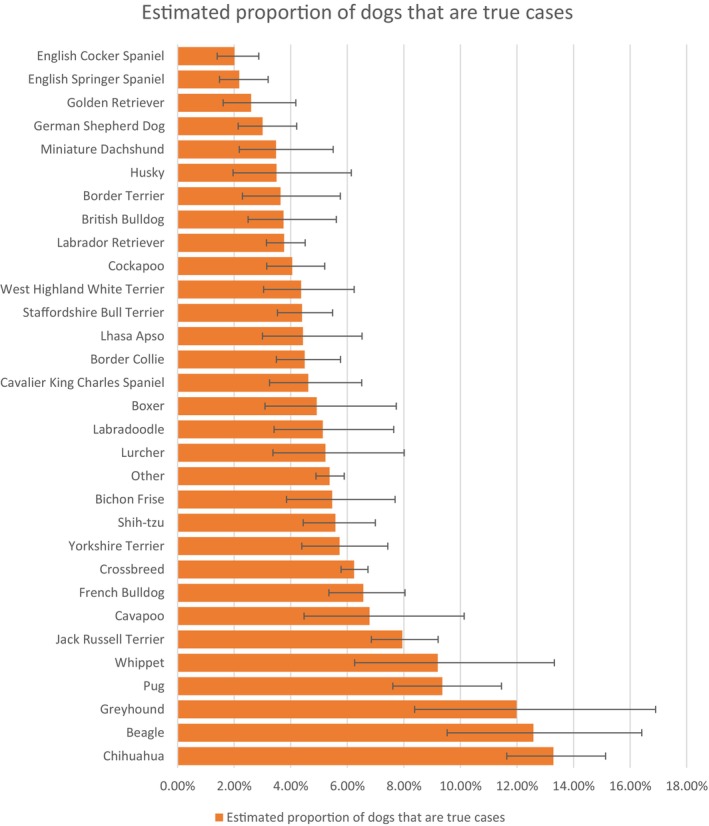
Annual proportional (%) nail clipping in common dog breeds under primary veterinary care in the VetCompass Programme in the United Kingdom in 2019. The horizontal bars represent 95% confidence intervals.

Text searches of the overall study population of 2,250,417 dogs under primary veterinary care at 1224 clinics participating in the VetCompass Programme in 2019 yielded 260,024 (11.56%) candidate nail clipping cases. Manual checking of a random sample of 4996 candidate cases (1.92% sampling proportion of candidates) identified 2440 confirmed nail clipping cases during 2019 from a notional 43,239 study population (*i.e*. 1.92% sampling proportion of study population). After accounting for the subsampling protocol, the estimated annual proportional (prevalence) nail clipping in dogs overall was 5.64% (95% CI 5.43 to 5.86). Breeds with the highest annual proportional nail clipping cases were Chihuahua (13.29%, 95% CI 11.64 to 15.13), beagle (12.58%, 95% CI 9.53 to 16.41), Greyhound (11.98%, 95% CI 8.38 to 16.90) and Pug (9.36%, 7.61 to 11.45) (Fig 1).

Of the nail clipping cases (*n* = 2440) with data available on the variable, 1670 (68.44%) were purebred, 1203 (49.30%) were female, and 1031 (42.25%) were neutered. The median age for cases was 5.07 years (IQR: 2.27 to 9.24, range 0.17 to 19.11). The median adult bodyweight was 10.99 kg (IQR: 8.50 to 24.70, range 1.90 to 72.00). Of the 2440 cases, the most presented breeds were Crossbreed (*n* = 614. 25.16%), Chihuahua (194, 7.95%), Jack Russell terrier (161, 6.60%) and Labrador retriever (112, 4.59%) (Table S1).

Of the dogs that were not nail clipping cases with data available on the variable, 550,761 (69.20%) were purebred, 377,869 (47.48%) were female, and 346,262 (43.51%) were neutered. The median age was 5.28 years (IQR: 2.27 to 9.04, range 0.003 to 24.91). The median adult bodyweight was 14.00 kg (IQR: 8.50 to 24.70, range 1.50 to 106.00). The most common breeds in the non‐case dogs were Crossbred (*n* = 188,306, 23.66%), Labrador retriever (56,208, 7.06%), Jack Russell terrier (35,311, 4.44%), English cocker spaniel (35,207, 4.42%) and Staffordshire Bull terrier (34,186, 4.30%) (Table S1).

### Risk factors for being a nail clipping case

Nine variables (breed, breed purity, Kennel Club breed group, skull conformation, chondrodystrophy, coat length, age, sex and neuter status) were liberally associated with nail clipping in univariable logistic regression modelling and were evaluated using multivariable logistic regression modelling (Tables S1, S2 and S3). The final breed‐focused multivariable model retained three risk factors: breed, age and sex. No biologically relevant interactions were detected in the final model. The final model showed no evidence of poor model fit (Hosmer–Lemeshow test statistic: P = 0.288).

After accounting for the effects of the other variables, 11 breeds showed increased odds of nail clipping compared with crossbreed dogs. The breeds with highest odds for nail clipping were Chihuahua (OR 2.21, 95% CI 1.88 to 2.60), Beagle (OR 2.09, 95% CI 1.54 to 2.83), Greyhound (OR 2.02, 95% CI 1.37 to 2.97), Pug (OR 1.84, 95% CI 1.46 to 2.32) and Whippet (OR 1.80, 95% CI 1.19 to 2.71). Two breeds showed lower odds of nail clipping compared with crossbreds: English springer spaniel (OR 0.42, 95% CI 0.28 to 0.62) and English cocker spaniel (OR 0.38, 95% CI 0.28 to 0.52). Compared with dogs aged 12 years or older, dogs aged 1 to 2 years showed the highest odds of nail clipping, whereas dogs aged under 1 year showed the lowest (Table 1).

**Table 1:** Breed‐focused multivariable logistic regression results for demographic risk factors for being a nail clipping case during 2019 in dogs under primary veterinary care in the VetCompass Programme in the United Kingdom

Variable	Category	Non‐case no. (%)	Case no. (%)	Odds ratio	95% CI	Category P‐value	Variable P‐value
Breed	Crossbreed	188,306 (23.66)	614 (25.16)	Base			<0.001
	Chihuahua	26,402 (3.32)	194 (7.95)	2.21	1.88 to 2.60	<0.001	
	Beagle	6493 (0.82)	45 (1.84)	2.09	1.54 to 2.83	<0.001	
	Greyhound	4204 (0.53)	27 (1.11)	2.02	1.37 to 2.97	<0.001	
	Pug	13,538 (1.70)	83 (3.40)	1.84	1.46 to 2.32	<0.001	
	Whippet	4085 (0.51)	24 (0.98)	1.80	1.19 to 2.71	0.005	
	Jack Russell terrier	35,311 (4.44)	161 (6.60)	1.44	1.21 to 1.72	<0.001	
	English Bulldog	8179 (1.03)	37 (1.52)	1.36	0.97 to 1.90	0.071	
	Cavapoo	4827 (0.61)	21 (0.86)	1.33	0.86 to 2.05	0.206	
	French Bulldog	23,570 (2.96)	86 (3.52)	1.10	0.87 to 1.38	0.418	
	Bichon frise	8649 (1.09)	30 (1.23)	1.06	0.74 to 1.53	0.750	
	Lurcher	5615 (0.71)	19 (0.78)	1.04	0.66 to 1.64	0.865	
	Miniature dachshund	8785 (1.10)	28 (1.15)	0.98	0.67 to 1.43	0.908	
	Shih‐tzu	23,446 (2.95)	70 (2.87)	0.91	0.71 to 1.17	0.454	
	Labradoodle	7551 (0.95)	22 (0.90)	0.88	0.58 to 1.35	0.569	
	Husky	6276 (0.79)	18 (0.74)	0.87	0.54 to 1.39	0.555	
	Other	132,491 (16.65)	369 (15.12)	0.86	0.76 to 0.98	0.027	
	Lhasa apso	8589 (1.08)	24 (0.98)	0.86	0.57 to 1.29	0.470	
	Yorkshire terrier	18,797 (2.36)	52 (2.13)	0.86	0.65 to 1.14	0.286	
	Border Collie	22,638 (2.84)	61 (2.50)	0.84	0.65 to 1.09	0.194	
	Boxer	6334 (0.80)	17 (0.70)	0.82	0.51 to 1.33	0.423	
	Cavalier King Charles spaniel	12,645 (1.59)	30 (1.23)	0.73	0.51 to 1.05	0.093	
	West Highland White terrier	12,533 (1.57)	28 (1.15)	0.72	0.49 to 1.05	0.084	
	Staffordshire Bull terrier	34,186 (4.30)	75 (3.07)	0.69	0.54 to 0.87	0.002	
	Cockapoo	25,427 (3.19)	58 (2.38)	0.68	0.52 to 0.90	0.006	
	Labrador retriever	56,208 (7.06)	112 (4.59)	0.62	0.50 to 0.75	<0.001	
	Border terrier	9175 (1.15)	17 (0.70)	0.58	0.36 to 0.94	0.027	
	German Shepherd dog	17,398 (2.19)	32 (1.31)	0.56	0.39 to 0.80	0.001	
	Golden retriever	9823 (1.23)	16 (0.66)	0.50	0.31 to 0.83	0.007	
	English Springer spaniel	19,203 (2.41)	26 (1.07)	0.42	0.28 to 0.62	<0.001	
	English Cocker spaniel	35,207 (4.42)	44 (1.80)	0.38	0.28 to 0.52	<0.001	
Age	>12.0	83,701 (10.52)	202 (8.28)				<0.001
	<1.0	84,524 (10.62)	207 (8.48)	0.98	0.80 to 1.19	0.800	
	1.0 to <2.0	90,883 (11.42)	374 (15.33)	1.61	1.35 to 1.92	<0.001	
	2.0 to <4.0	140,071 (17.60)	426 (17.46)	1.18	0.99 to 1.40	0.061	
	4.0 to <6.0	121,155 (15.22)	391 (16.02)	1.25	1.06 to 1.49	0.010	
	6.0 to <8.0	106,097 (13.33)	366 (15.00)	1.37	1.15 to 1.62	<0.001	
	8.0 to <10.0	90,809 (11.41)	274 (11.23)	1.23	1.02 to 1.47	0.029	
	10.0 to 12.0	72,026 (9.05)	195 (7.99)	1.12	0.92 to 1.37	0.256	
	Unrecorded	6625 (0.83)	5 (0.20)	0.32	0.13 to 0.78	0.013	
Sex	Female	377,869 (47.48)	1203 (49.30)				0.001
	Male	410,560 (51.58)	1234 (50.57)	0.94	0.87 to 1.02	0.143	
	Unrecorded	7462 (0.94)	3 (0.12)	0.14	0.05 to 0.44	<0.001	

Column percentages are shown in brackets. Total of 2440 cases and 795,891 non‐cases.

CI Confidence interval

As described in the Methods, breed‐derived variables were introduced individually to replace *breed* in the final breed‐focused model. Purebred dogs had lower odds of nail clipping compared to general crossbred dogs (OR 0.94, 95% CI 0.85 to 1.03). Dogs in the Kennel Club Hound and Toy breed groups had higher odds of nail clipping compared to the dogs that were non‐Kennel Club recognised breeds. Dogs with medium coat length had lower odds of nail clipping compared to those with short coat length (OR 0.67, 95% CI 0.59 to 0.75). Chondrodystrophic dogs exhibited higher odds of nail clipping compared to non‐chondrodystrophic dogs (OR 1.44, 95% CI 1.31 to 1.59). Dogs weighing <10.0 kg had higher odds of overgrown nails compared to dogs weighing 40.0 kg or more (OR 1.84, 95% CI 1.36 to 2.49) (Table 2).

**Table 2:** Descriptive and multivariable binary logistic regression results for breed‐derived risk factors and bodyweight checked for nail clipping cases during 2019 in dogs under primary veterinary care in the VetCompass programme in the United Kingdom

Variable	Category	Non‐case no. (%)	Case no. (%)	Odds ratio	95% CI	Category P‐value	Variable P‐value
Breed purity	General crossbred	188,306 (23.66)	614 (25.16)	Base			0.052
	Purebred	550,761 (69.20)	1670 (68.44)	0.935	0.85 to 1.03	0.153	
	Designer crossbreed	51,713 (6.50)	152 (6.23)	0.875	0.73 to 1.05	0.142	
	Unrecorded	5111 (0.64)	4 (0.16)	0.319	0.12 to 0.86	0.024	
Kennel Club recognised breed	Not recognised	254,787 (32.01)	799 (32.75)	Base			0.087
	Recognised	535,993 (67.35)	1637 (67.09)	0.981	0.90 to 1.07	0.651	
	Unrecorded	5111 (0.64)	4 (0.16)	0.331	0.12 to 0.89	0.029	
Kennel Club breed group	Not Kennel Club recognised breed	254,787 (32.01)	799 (32.75)	Base			<0.001
	Hound	32,868 (4.13)	168 (6.89)	1.635	1.38 to 1.93	<0.001	
	Toy	95,467 (11.99)	438 (17.95)	1.451	1.29 to 1.63	<0.001	
	Terrier	104,308 (13.11)	330 (13.52)	1.044	0.92 to 1.19	0.52	
	Utility	93,204 (11.71)	282 (11.56)	0.954	0.83 to 1.09	0.50	
	Working	27,961 (3.51)	66 (2.70)	0.743	0.58 to 0.96	0.02	
	Pastoral	46,528 (5.85)	107 (4.39)	0.74	0.61 to 0.91	0.00	
	Gundog	135,657 (17.04)	246 (10.08)	0.584	0.51 to 0.67	<0.001	
	Unrecorded	5111 (0.64)	4 (0.16)	0.333	0.12 to 0.89	0.03	
Skull conformation	Mesocephalic	385,944 (48.49)	966 (39.59)	Base			<0.001
	Brachycephalic	145,101 (18.23)	619 (25.37)	1.673	1.51 to 1.85	<0.001	
	Dolichocephalic	71,429 (8.97)	237 (9.71)	1.321	1.15 to 1.52	<0.001	
	Unrecorded	193,417 (24.30)	618 (25.33)	1.278	1.16 to 1.41	<0.001	
Coat length	Short	284,391 (35.73)	915 (37.50)	Base			<0.001
	Long	65,717 (8.26)	187 (7.66)	0.881	0.75 to 1.03	0.120	
	Medium	171,385 (21.53)	365 (14.96)	0.666	0.59 to 0.75	<0.001	
	Hairless	276 (0.03)	0 (0.00)	0	0.00‐.	1.00	
	Unrecorded	274,122 (34.44)	973 (39.88)	1.102	1.01 to 1.21	0.040	
Chondystrophy	Not chondystrophic	265,373 (33.34)	653 (26.76)			<0.001	
	Chondystrophic	285,342 (35.85)	1016 (41.64)	1.443	1.31 to 1.59	<0.001	
	Unrecorded	245,176 (30.81)	771 (31.60)	1.274	1.15 to 1.42	<0.001	
Bodyweight (kg)	≥40	17,977 (2.26)	44 (1.80)				<0.001
	<10	183,476 (23.05)	825 (33.81)	1.84	1.36 to 2.49	<0.001	
	10.0 to <20.0	167,168 (21.00)	488 (20.00)	1.19	0.88 to 1.63	0.263	
	20.0 to <40.0	171,178 (21.51)	471 (19.30)	1.14	0.84 to 1.56	0.404	
	Unrecorded	256,092 (32.18)	612 (25.08)	0.87	0.64 to 1.20	0.398	

Column percentages are shown in brackets. Total of 2440 cases and 795,891 non‐cases.

CI Confidence interval

### Clinical

**FIG 2:**
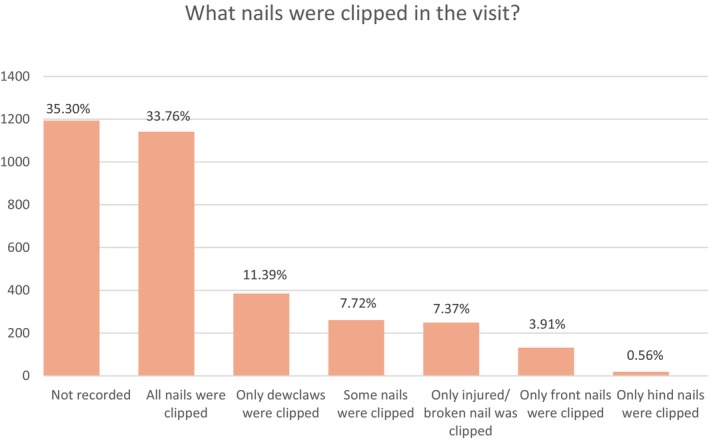
The nails that were clipped during 3380 nail clipping events recorded in the VetCompass Programme in dogs under primary veterinary care during 2019.

**FIG 3:**
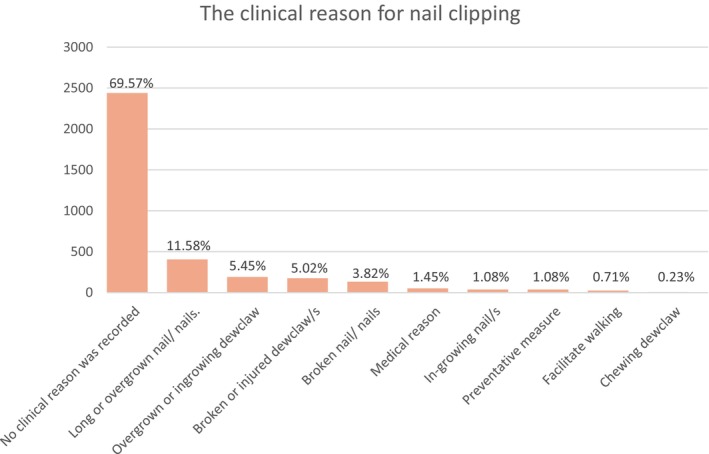
Clinical justifications for 3380 nail clipping events recorded in the VetCompass Programme in dogs under primary veterinary care during 2019.

There were 3380 nail clipping events recorded across the random sample of 2440 nail clipping cases during 2019. The median number of annual nail clipping events per nail clipping case was 1 (IQR 1 to 2, range 1 to 11), with 54.53% (*n* = 1843) nail clipping cases having only one recorded nail clipping event in 2019. Nail clipping was stated in the EHR as the primary reason for the veterinary visit in 59.40% (2009/3380) events. The motivation for the nail clipping was on request by the owners in 55.71% (*n* = 1883) events, as a part of routine checkup or care plan in 31.86% (*n* = 1077) events and primarily for a clinical reason in 12.43% (*n* = 420) events. Every nail was clipped in 33.76% (*n* = 1141) events, an unspecified number of nails were clipped in 7.72% (*n* = 261) events, while nail clipping was restricted to the dewclaw(s) alone in 11.39% (*n* = 385) events, injured or broken claw in 7.37% (*n* = 249) events, front claw in 3.19% (*n* = 132) and hind claw in 0.56% (*n* = 19) events (Fig 2). The most common clinical justifications for nail clipping among 3380 nail clipping events included overgrown or ingrown nails in 12.66% (*n* = 444), broken claws or dewclaws in 8.84% (*n* = 310), medical reasons in 1.45% (*n* = 51), preventive measures in 1.08% (*n* = 38) and to facilitate easier walking in 0.71% (*n* = 25). No specific clinical justifications for the nail clipping were recorded in 69.57% (*n* = 2439) events, and in some events, more than one clinical reason for nail clipping was recorded (Fig 3).

## DISCUSSION

The current study reported 1‐year proportional (*i.e*. prevalence) nail clipping at 5.64% in dogs attending primary veterinary clinics. The prevalence of overgrown nails in the UK dog population was previously reported as 5.52% (O'Neill et al., 2021) which is very similar to the current value, while torn or broken nails were previously reported in the United States in 5.66% of purebreds and 4.83% of crossbreds (Forsyth et al., 2023). The high prevalence data reported in the current study suggest substantial welfare value from greater emphasis on teaching good canine nail care in veterinary and veterinary nursing education. Training at both undergraduate and postgraduate levels should ensure professionals are confident in performing nail clipping procedures safely and effectively. Structured teaching and continuing professional development on nail clipping could help embed this routine task more firmly within evidence‐based practice. The current results can promote improved animal welfare by guiding veterinary clinics towards better resource allocation for this commonly requested procedure. Veterinary clinics may consider prioritising nail care that could include establishing dedicated nurse‐led nail care clinics as a strategic response and ensuring that routine nail maintenance is more readily accessible to all pet owners. Protocols or guidelines could be developed and made available in the clinics for client education and to remind the veterinary staff about the importance of good canine nail care. While many clinics already offer nail care as a routine add‐on service for dogs undergoing sedation or anaesthesia for other reasons, wider adoption of this approach could further optimise time and resources, while also allowing for improved precision and control during the procedure (Karas, 1999).

The current study identified breed as a strong risk factor for nail clipping. Chihuahua (OR 2.21, prevalence annual 13.29%), beagle (OR 2.09, 12.58%) and Greyhound (OR 2.02, 11.98%) showed the highest relative and absolute risk of nail clipping compared to Crossbreed dogs. The prevalence of overgrown nails in Chihuahuas was previously reported at 4.00% (O'Neill, Pegram, et al., 2020) which is lower than the current result. The predisposition to nail clipping identified for Beagles in the current study aligns closely with a recent study that ranked overgrown nails as the third most diagnosed condition in Beagles (11.61%) (O'Neill et al., 2025). A predisposition in Greyhounds concurs with previous evidence where overgrown nails were reported as the second most commonly recorded disorder in Greyhounds with a prevalence of 11.1% (O'Neill et al., 2019) and also research reporting the incidence of overgrown nails in the hound breed group more generally at 41.2% (Olsson Wiberg, 2024). Interestingly, six of the eight breeds with the highest odds of nail clipping were from the hound or toy group. Differences in nail characteristics such as quality, width and hardness between breeds as well as differing behavioural drives for exercise, might contribute to variations in nail wear and the resulting need for veterinary nail care. A naturally high behavioural drive for extensive exercise in hound dogs originally bred for hunting may contribute to a predisposition for nail clipping and overgrown nails when these exercise needs remain unmet and frustrated during more sedentary lives as companion animals (Elliott et al., 2010; Pickup et al., 2017). In a study surveying the activity level in different dog breeds in the UK, dogs from the Toy, Hound, and Terrier groups were less likely to receive at least 30 minutes of exercise per day compared to those in the Gundog, Pastoral and Working groups (Pickup et al., 2017), possibly explaining why these breeds are at a higher risk for overgrown nails and decreased levels of natural nail wearing. For Toy breeds, selective breeding towards miniaturisation that diverges strongly from the natural size of ancestral wild dogs could result in reduced gravitational pressure and friction, which might further reduce nail wear‐down (Worboys et al., 2018). In addition to the intrinsically higher risk of nail overgrowth, smaller dogs may also receive higher levels of veterinary presentation for routine nail care because of logistical factors whereby veterinary visits and transportation of smaller dogs are reported to be easier than for larger breed dogs (Park et al., 2021). The current evidence that artificial selection in dogs towards extreme body sizes has predisposed the extreme dog to nail overgrowth is supported by a previous report that 78% of dog breeds with a bodyweight of 21 to 30 kg typically of an average‐sized dog have healthier nails and fewer cases of overgrown nails compared to the other weight groups (Olsson Wiberg, 2024).

Age was significantly associated with the odds of being a nail clipping case in the current study, with dogs aged 1 to 2 years demonstrating the highest odds. Predisposition in young adult dogs in the current study does not align with some previously proposed theories that older dogs should need more frequent nail clipping because of reduced exercise levels along with their rising ageing‐related levels of musculoskeletal disease (Orpet & Welsh, 2010; Wheatley, 2018). Younger dogs have been reported to have healthier claws in general but to experience more incidents of torn or broken claws, which could partially explain the current predisposition in young adult dogs (Olsson Wiberg, 2024). It is also possible that increased odds in younger dogs could reflect their more novice owners seeking greater veterinary guidance on performing basic nail care procedures during early phases of dog ownership but followed by reducing numbers of veterinary nail care visits as the owners gain more experience of dog husbandry and are more confident to undertake nail clipping themselves at home (Park et al., 2021). Additionally, rates of nail growth are reported to decrease with age, leading to an intrinsically reduced risk of overgrowth over the lifetime of each dog, although that study was conducted in a laboratory setting and may poorly generalise to the wider dog population because of widely differing factors such as environment, exercise level and diet (Orentreich et al., 1979). However, regardless of differing odds across the age groups, nail clipping was still common across all age groups, and therefore owners and veterinarians should prioritise good nail care and maintenance as a recurring lifetime goal for dogs of all ages.

In the current study, 75.20% of the nail clipping cases had just a single nail clipping event recorded in 2019, while 16.40% had two nail clipping events. This aligns with a previous survey of dog owners, which reported that owners considered their dogs required nail clipping at least twice a year, although one‐third of the owners reported their dogs not to require any nail clipping (Edwards et al., 2022). The current results also reported nail clipping as the primary reason for veterinary visits in 59.40% of the events, suggesting that many owners rely heavily on veterinary care to maintain nail health in their dogs. Consequently, veterinary practices could improve their contribution to overall canine welfare by providing greater focus on nail clipping services by, for example, providing training and high‐quality equipment for their veterinary professionals to promote better procedure performance (Riemer et al., 2021).

The dewclaw(s) alone was(were) clipped in 11.39% of the nail clipping events, while broken, injured, overgrown and ingrown dewclaws represented 10.47% of the documented clinical justifications for nail clipping. This finding of high contribution of dewclaw issues to the wider veterinary nail clipping caseload is supported by previous literature that also highlighted high levels of overgrowing and injury risk for dewclaws (Covaşǎ & Şerban, 2021; Hughes & Soloman‐Kretay, 2007; Miller et al., 2012) and may suggest that special priority should be given to dewclaw care by owners and veterinarians. Routine elective dewclaw amputation, often when puppies are around 3 days old (Hoskins, 2001), raises many ethical issues and is widely considered a potential form of mutilation (Mills et al., 2016), with poor evidence on any long‐term welfare implications. The current study did not explicitly explore the effects of dewclaw amputation, so there remains a need for specific epidemiological research on dewclaw amputation before formal evidence‐based clinical recommendations can be made (Diesel et al., 2010; Tillson, 2024).

While access to the current large data set of contemporaneously recorded clinical records in VetCompass Programme offered many advantages including the depth of information in the free‐text clinical notes and for high statistical power in the analyses (O'Neill et al., 2014), the study still had some limitations. These clinical records were secondary data not initially intended for research, so many of these notes did not record the level of detail and specificity on nail clipping that prospective primary research might achieve (Jones‐Diette et al., 2017). The nail clipping events examined in this study included only those events conducted under primary veterinary care and therefore this limits generalisation of the current results to other contexts of nail clipping such as events performed by groomers and by pet owners themselves. This may also lead to underestimation of total clipping events within the overall lives of the dogs in the current dataset. The job status (*e.g*. veterinarian, veterinary nurse, etc.) of the person performing the nail clipping was not routinely recorded in the clinical records, so this information was unavailable for the analysis. Inclusion of nail clipping data from groomers and owners in future studies could offer a more comprehensive understanding of the factors influencing canine welfare during nail clipping. Such an expanded dataset would allow for the comparison of different approaches and environments, potentially highlighting best practices and areas needing improvement across different care contexts.

In conclusion, this study revealed substantial demand for veterinary nail care among dog owners, with 5.64% of all dogs under veterinary care receiving at least one nail clipping event each year. Certain breeds, including Chihuahua, beagle and Greyhound, were shown to be predisposed to nail clipping, further highlighting breed and dog conformation as key drivers of overall dog health and welfare. The current findings underscore the importance of tailored veterinary guidance and breed‐specific considerations in promoting optimal nail health and contributing to better overall canine welfare. By identifying at‐risk groups and quantifying the frequency of nail‐related issues, this study supports more proactive veterinary monitoring and owner education, which together may reduce preventable complications such as overgrown nails, ingrown claws and associated pain or infections. Nail clipping should be promoted as a veterinary care intervention of high importance and included explicitly as a key component of undergraduate veterinary and veterinary nursing educational curricula. Further research is needed to better understand the mechanical processes underlying the factors identified here contributing to nail clipping frequency.

## Author contributions

**L. A. Ahmed:** Writing – original draft; writing – review and editing; formal analysis; conceptualization; data curation; visualization. **M. Somarriba:** Conceptualization; writing – review and editing; supervision. **D. C. Brodbelt:** Data curation; software; writing – review and editing; validation. **D. B. Church:** Data curation; validation. **D. G. O’Neill:** Conceptualization; methodology; writing – review and editing; supervision; data curation; funding acquisition; visualization; project administration; validation.

## Funding information

This study was supported at the RVC by an award from the Kennel Club Charitable Trust and Agria Pet Insurance. Neither the Kennel Club Charitable Trust, Agria Pet Insurance nor the Kennel Club had any input in the design of the study, the collection, analysis and interpretation of data or in writing the manuscript.

## Conflict of interest statement

The authors have no conflicts of interest to declare.

## Supporting information


Table S1.

Table S2.

Table S3.

## Data Availability

The data sets generated during and/or analysed during the current study are available at Figshare – DOI 10.6084/m9.figshare.27135756.

## References

[jsap70002-bib-0001] Asher, L. , Buckland, E. , Phylactopoulos, C.L. , Whiting, M. , Abeyesinghe, S. & Wathes, C. (2011) Estimation of the number and demographics of companion dogs in the UK. BMC Veterinary Research, 7, 74. 22112367 10.1186/1746-6148-7-74 PMC3305510

[jsap70002-bib-0002] Carbonell Buj, E. & Farrell, M. (2019) Permanent cessation of nail growth using multiple nail plate avulsions and phenolisation in a dog. Veterinary Record Case Reports, 7, e000757.

[jsap70002-bib-0003] Covaşǎ, C.T. & Şerban, C. (2021) Hind limb first digit (dewclaw) in dog: morphologic and X‐ray investigation in relation with genetic and surgical aspects. Revista Română de Medicină Veterinară, 31, 33–38.

[jsap70002-bib-0004] Dean, A. , Sullivan, K. & Soe, M. (2022) OpenEpi: open source epidemiologic statistics for public health. Available from: https://www.openepi.com/Menu/OE_Menu.htm [Accessed 18th December].

[jsap70002-bib-0005] Diesel, G. , Pfeiffer, D. , Crispin, S. & Brodbelt, D. (2010) Risk factors for tail injuries in dogs in Great Britain. Veterinary Record, 166, 812–817. 20581358 10.1136/vr.b4880

[jsap70002-bib-0006] Edwards, P.T. , Smith, B.P. , McArthur, M.L. & Hazel, S.J. (2022) Puppy pedicures: exploring the experiences of Australian dogs to nail trims. Applied Animal Behaviour Science, 255, 105730. Available from: 10.1016/j.applanim.2022.105730

[jsap70002-bib-0007] Elliott, R. , Toribio, J.‐A.L.M.L. & Wigney, D. (2010) The greyhound adoption program (GAP) in Australia and New Zealand: a survey of owners' experiences with their greyhounds one month after adoption. Applied Animal Behaviour Science, 124, 121–135. Available from: 10.1016/j.applanim.2010.02.006

[jsap70002-bib-0008] Forsyth, K.K. , McCoy, B.M. , Schmid, S.M. , Promislow, D.E. , Snyder‐Mackler, N. , Consortium, D. et al. (2023) Lifetime prevalence of owner‐reported medical conditions in the 25 most common dog breeds in the dog aging project pack. Frontiers in Veterinary Science, 10, 1140417. 38026653 10.3389/fvets.2023.1140417 PMC10655140

[jsap70002-bib-0009] Hoskins, J.D. (2001) Veterinary pediatrics: dogs and cats from birth to six months. 3rd edition. Philadelphia, PA: W.B. Saunders. p. 367.

[jsap70002-bib-0038] Hosmer, D.W. , Jr., Lemeshow, S. & Sturdivant, R.X. (2013) Assessing the fit of the model. In: Applied logistic regression. 3rd edition. Hoboken, NJ: Wiley. p. 153–226.

[jsap70002-bib-0010] Hughes, A. & Soloman‐Kretay, J. (2007) Basic animal care procedures. In: BSAVA manual of practical animal care. Gloucester: British Small Animal Veterinary Association, pp. 14–31.

[jsap70002-bib-0039] IBM Corp . (2024). IBM SPSS Statistics for Windows, Version 29.0 [Software]. IBM Corp. https://www.ibm.com/products/spss.

[jsap70002-bib-0011] Jackson, H.A. & Marsella, R. (2012) An approach to diseases of the claws and claw folds. In: BSAVA manual of canine and feline dermatology. Gloucester: British Small Animal Veterinary Association, p. 121.

[jsap70002-bib-0012] Jones‐Diette, J. , Robinson, N.J. , Cobb, M. , Brennan, M.L. & Dean, R.S. (2017) Accuracy of the electronic patient record in a first opinion veterinary practice. Preventive Veterinary Medicine, 148, 121–126. Available from: 10.1016/j.prevetmed.2016.11.014 28233582

[jsap70002-bib-0013] Karas, A.Z. (1999) Sedation and chemical restraint in the dog and cat. Clinical Techniques in Small Animal Practice, 14, 15–26. Available from: 10.1016/S1096-2867(99)80023-1 10193042

[jsap70002-bib-0014] Kirkwood, B.R. & Sterne, J.A.C. (2003) Essential medical statistics, 2nd edition. Oxford: Blackwell Science.

[jsap70002-bib-0015] Miller, W.H. , Griffin, C.E. & Campbell, K.L. (2012) 'Disorders of the claws. In: Muller and Kirk's small animal dermatology, 7th edition. St. Louis: Elsevier Health Sciences, pp. 1483–1499.

[jsap70002-bib-0016] Mills, K.E. , von Keyserlingk, M.A. & Niel, L. (2016) A review of medically unnecessary surgeries in dogs and cats. Journal of the American Veterinary Medical Association, 248, 162–171. 26720081 10.2460/javma.248.2.162

[jsap70002-bib-0018] O'Neill, D.G. , Church, D.B. , McGreevy, P.D. , Thomson, P.C. & Brodbelt, D.C. (2014) Approaches to canine health surveillance. Canine Genetics and Epidemiology, 1, 1–13. 26401319 10.1186/2052-6687-1-2 PMC4574389

[jsap70002-bib-0019] O'Neill, D.G. , James, H. , Brodbelt, D.C. , Church, D.B. & Pegram, C. (2021) Prevalence of commonly diagnosed disorders in UK dogs under primary veterinary care: results and applications. BMC Veterinary Research, 17, 69. Available from: 10.1186/s12917-021-02775-3 33593363 PMC7888168

[jsap70002-bib-0020] O'Neill, D.G. , Packer, R. , Lobb, M. , Church, D.B. , Brodbelt, D.C. & Pegram, C. (2020) Demography and commonly recorded clinical conditions of Chihuahuas under primary veterinary care in the UK in 2016. BMC Veterinary Research, 16, 1–14. 32046714 10.1186/s12917-020-2258-1 PMC7014602

[jsap70002-bib-0021] O'Neill, D.G. , Pegram, C. , Crocker, P. , Brodbelt, D.C. , Church, D.B. & Packer, R.M.A. (2020) Unravelling the health status of brachycephalic dogs in the UK using multivariable analysis. Scientific Reports, 10, 17251. Available from: 10.1038/s41598-020-73088-y 33057051 PMC7560694

[jsap70002-bib-0022] O'Neill, D.G. , Rooney, N.J. , Brock, C. , Church, D.B. , Brodbelt, D.C. & Pegram, C. (2019) Greyhounds under general veterinary care in the UK during 2016: demography and common disorders. Canine Genetic Epidemiology, 6, 4. Available from: 10.1186/s40575-019-0072-5 PMC6547581 31179010

[jsap70002-bib-0023] O'Neill, D.G. , Schiksnis, M.R. , Brodbelt, D.C. , Church, D.B. , Goldberg, S. & Engdahl, K.S. (2025) Beagles kept as companion animals in the UK – demography, disorders and mortality. Companion Animal Health and Genetics, 12, 1. 10.1186/s40575-024-00140-9 PMC11827713 42135833

[jsap70002-bib-0017] Olsson Wiberg, I.J. (2024) The influence of risk factors on the health of dogs' paws and the importance of clinical evaluation of the paw in clinical practice. Unpublished Thesis.

[jsap70002-bib-0024] Orentreich, N. , Markofsky, J. & Vogelman, J.H. (1979) The effect of aging on the rate of linear nail growth. Journal of Investigative Dermatology, 73, 126–130. Available from: 10.1111/1523-1747.ep12532799 448171

[jsap70002-bib-0025] Orpet, H. & Welsh, P. (2010) Contemporary veterinary nursing. In: Handbook of veterinary nursing, 2nd edition. Oxford: John Wiley & Sons, pp. 72–73.

[jsap70002-bib-0026] Park, R.M. , Gruen, M.E. & Royal, K. (2021) Association between dog owner demographics and decision to seek veterinary care. Veterinary Sciences, 8, 7. 33466270 10.3390/vetsci8010007 PMC7824748

[jsap70002-bib-0027] Pickup, E. , German, A.J. , Blackwell, E. , Evans, M. & Westgarth, C. (2017) Variation in activity levels amongst dogs of different breeds: results of a large online survey of dog owners from the UK. Journal of Nutritional Science, 6, e10. 28620485 10.1017/jns.2017.7 PMC5465859

[jsap70002-bib-0028] Riemer, S. , Heritier, C. , Windschnurer, I. , Pratsch, L. , Arhant, C. & Affenzeller, N. (2021) A review on mitigating fear and aggression in dogs and cats in a veterinary setting. Animals, 11, 158. 33445559 10.3390/ani11010158 PMC7826566

[jsap70002-bib-0029] Skipper, A.M. , Packer, R.M. & O'Neill, D.G. (2024) “Maybe we should think outside the box?” prioritisation of issues with UK not‐for‐profit canine health and welfare research funding using Delphi expert consensus and gap analysis. PLoS One, 19, e0313735. 39630686 10.1371/journal.pone.0313735 PMC11616890

[jsap70002-bib-0030] The Kennel Club . (2025) Breed information centre. Available from: https://www.thekennelclub.org.uk/search/breeds‐a‐to‐z [Accessed 9th February].

[jsap70002-bib-0031] The VeNom Coding Group . (2024) VeNom veterinary nomenclature. Available from: http://venomcoding.org [Accessed 12th November].

[jsap70002-bib-0032] Tillson, D.M. (2024) Digit amputation. In: Tillson, D.M. , (Ed.) Techniques in small animal soft tissue, orthopedic, and ophthalmic surgery. Hoboken, NJ: Wiley, pp. 189–195.

[jsap70002-bib-0033] VetCompass . (2025) VetCompass Programme. Available from: http://www.rvc.ac.uk/VetCOMPASS/ [Accessed 25th January].

[jsap70002-bib-0034] Wheatley, S. (2018) Animal restraint for veterinary professionals. The Canadian Veterinary Journal, 59, 894.

[jsap70002-bib-0035] Worboys, M. , Strange, J.‐M. & Pemberton, N. (2018) Adopting breed. In: The invention of the modern dog: breed and blood in Victorian Britain. Baltimore, MD: Johns Hopkins University Press, pp. 45–60.

