# Supplementary material for: Epidemiology and clinical management of nail clipping in dogs under UK primary veterinary care
Source: J Small Anim Pract. 2025 Aug 5;66(12):925–33. doi: 10.1111/jsap.70002 (PMC12686259; doi:10.1111/jsap.70002)
Supplement: Supplementary file 1 — Table S1. [file JSAP-66-925-s001.pdf]

Supplementary Table 1: Descriptive and univariable binary logistic regression results for breed as a risk factor for nail clipping during 2019 in dogs under primary veterinary care in the VetCompass programme in the UK.

Breed	Non-case No. (%)	Case No. (%)	Odds Ratio	95% CI*	Category P-value	Variable P-value
Crossbreed	188306 (23.66)	614 (25.16)	Base			<.001
Chihuahua	26402 (3.32)	194 (7.95)	2.25	1.92-2.65	<.001	
Beagle	6493 (0.82)	45 (1.84)	2.13	1.57-2.88	<.001	
Greyhound	4204 (0.53)	27 (1.11)	1.97	1.34-2.90	<.001	
Pug	13538 (1.70)	83 (3.40)	1.88	1.49-2.37	<.001	
Whippet	4085 (0.51)	24 (0.98)	1.80	1.20-2.71	0.005	
Jack Russell Terrier	35311 (4.44)	161 (6.60)	1.40	1.18-1.66	<.001	
English Bulldog	8179 (1.03)	37 (1.52)	1.39	1.00-1.94	0.054	
Cavapoo	4827 (0.61)	21 (0.86)	1.33	0.86-2.06	0.195	
French Bulldog	23570 (2.96)	86 (3.52)	1.12	0.89-1.40	0.330	
Bichon Frise	8649 (1.09)	30 (1.23)	1.06	0.74-1.54	0.741	
Lurcher	5615 (0.71)	19 (0.78)	1.04	0.66-1.64	0.874	
Miniature Dachshund	8785 (1.10)	28 (1.15)	0.98	0.67-1.43	0.906	
Shih-tzu	23446 (2.95)	70 (2.87)	0.92	0.72-1.17	0.485	
Labradoodle	7551 (0.95)	22 (0.90)	0.89	0.58-1.37	0.604	
Husky	6276 (0.79)	18 (0.74)	0.88	0.55-1.41	0.592	
Lhasa Apso	8589 (1.08)	24 (0.98)	0.86	0.57-1.29	0.459	
Other	132491 (16.65)	369 (15.12)	0.85	0.75-0.97	0.017	
Yorkshire Terrier	18797 (2.36)	52 (2.13)	0.85	0.64-1.13	0.256	
Border Collie	22638 (2.84)	61 (2.50)	0.83	0.64-1.08	0.156	
Boxer	6334 (0.80)	17 (0.70)	0.82	0.51-1.33	0.429	
Cavalier King Charles Spaniel	12645 (1.59)	30 (1.23)	0.73	0.50-1.05	0.089	
Cockapoo	25427 (3.19)	58 (2.38)	0.70	0.53-0.92	0.009	
West Highland White Terrier	12533 (1.57)	28 (1.15)	0.69	0.47-1.00	0.051	
Staffordshire Bull Terrier	34186 (4.30)	75 (3.07)	0.67	0.53-0.86	0.001	
Labrador Retriever	56208 (7.06)	112 (4.59)	0.61	0.50-0.75	<.001	
Border Terrier	9175 (1.15)	17 (0.70)	0.57	0.35-0.92	0.022	
German Shepherd Dog	17398 (2.19)	32 (1.31)	0.56	0.40-0.81	0.002	
Golden Retriever	9823 (1.23)	16 (0.66)	0.50	0.30-0.82	0.006	
English Springer Spaniel	19203 (2.41)	26 (1.07)	0.42	0.28-0.62	<.001	
English Cocker Spaniel	35207 (4.42)	44 (1.80)	0.38	0.28-0.52	<.001	[1]

[1] Column percentages are shown in brackets.

*CI confidence interval

Total of 2440 cases and 795,891 non case
