# Supplementary material for: Epidemiology and clinical management of nail clipping in dogs under UK primary veterinary care
Source: J Small Anim Pract. 2025 Aug 5;66(12):925–33. doi: 10.1111/jsap.70002 (PMC12686259; doi:10.1111/jsap.70002)
Supplement: Supplementary file 2 — Table S2. [file JSAP-66-925-s003.pdf]

Supplementary table 2: Descriptive and univariable binary logistic regression results for non-breed-derived risk factors for nail clipping cases during 2019 in dogs under primary veterinary care in the VetCompass programme in the UK.

**Variable**	**Category**	**Non-case No. (%)**	**Case No. (%)**	**Odds ratio**	**95% CI***	**Category *P*-value**	**Variable *P*-value**
Age (years)	>12.0	83701 (10.52)	202 (8.28)	Base			<.001
	<1.0	84524 (10.62)	207 (8.48)	1.02	0.84-1.23	0.882	
	1.0-<2.0	90883 (11.42)	374 (15.33)	1.71	1.44-2.02	<.001	
	2.0-<4.0	140071 (17.60)	426 (17.46)	1.26	1.07-1.49	0.007	
	4.0-<6.0	121155 (15.22)	391 (16.02)	1.34	1.13-1.59	<.001	
	6.0-<8.0	106097 (13.33)	366 (15.00)	1.43	1.20-1.70	<.001	
	8.0-<10.0	90809 (11.41)	274 (11.23)	1.25	1.04-1.50	0.016	
	10.0-12.0	72026 (9.05)	195 (7.99)	1.12	0.92-1.37	0.253	
	Unrecorded	6625 (0.83)	5 (0.20)	0.31	0.13-0.76	0.010	
Sex	Female	377869 (47.48)	1203 (49.30)	Base			<.001
	Male	410560 (51.58)	1234 (50.57)	0.94	0.87-1.02	0.156	
	Unrecorded	7462 (0.94)	3 (0.12)	0.13	0.04-0.39	<.001	
Neuter	Entire	442167 (55.56)	1406 (57.62)	Base			<.001
	Neutered	346262 (43.51)	1031 (42.25)	0.936	0.86-1.02	0.109	
	Unrecorded	7462 (0.94)	3 (0.12)	0.126	0.04-0.39	<.001	
Bodyweight (Kg)	≥40	17977 (2.26)	44 (1.80)	Base			<.001
	<10	183476 (23.05)	825 (33.81)	1.84	1.36-2.49	<.001	
	10.0-<20.0	167168 (21.00)	488 (20.00)	1.19	0.88-1.62	0.260	
	20.0-<40.0	171178 (21.51)	471 (19.30)	1.12	0.88-1.53	0.460	
	Unrecorded	256092 (32.18)	612 (25.08)	0.98	0.72-1.33	0.880	[1]

[1] Column percentages are shown in brackets.
*CI confidence interval
Total of 2440 cases and 795,891 non case
