# Supplementary material for: Epidemiology and clinical management of nail clipping in dogs under UK primary veterinary care
Source: J Small Anim Pract. 2025 Aug 5;66(12):925–33. doi: 10.1111/jsap.70002 (PMC12686259; doi:10.1111/jsap.70002)
Supplement: Supplementary file 3 — Table S3. [file JSAP-66-925-s002.pdf]

Supplementary table 3: Descriptive and univariable logistic regression results for breed-related demographic risk factors checked for nail clipping cases during 2019 in dogs under primary veterinary care in the VetCompass programme in the UK.

**Variable**	**Category**	**Non-case No. (%)**	**Case No. (%)**	**Odds ratio**	**95% CI***	**Category P-value**	**Variable P-value**
Breed purity	General Crossbreed	188306 (23.66)	614 (25.16)	Base			0.017
	Designer crossbreed	51713 (6.50)	152 (6.23)	0.90	0.76-1.08	0.253	
	Purebred	550761 (69.20)	1670 (68.44)	0.93	0.85-1.02	0.124	
	Unrecorded	5111 (0.64)	4 (0.16)	0.24	0.09-0.64	0.004	
Kennel Club Recognised Breed	Not Recognised	254787 (32.01)	799 (32.75)	Base			0.020
	Recognised	535993 (67.35)	1637 (67.09)	0.97	0.90-1.06	0.541	
	Unrecorded	5111 (0.64)	4 (0.16)	0.25	0.09-0.67	0.006	
Kennel Club Breed Group	Not Recognised	254787 (32.01)	799 (32.75)	Base			<.001
	Hound	32868 (4.13)	168 (6.89)	1.63	1.38-1.93	<.001	
	Toy	95467 (11.99)	438 (17.95)	1.46	1.30-1.64	<.001	
	Terrier	104308 (13.11)	330 (13.52)	1.01	0.89-1.15	0.893	
	Utility	93204 (11.71)	282 (11.56)	0.97	0.84-1.11	0.606	
	Working	27961 (3.51)	66 (2.70)	0.75	0.59-0.97	0.027	
	Pastoral	46528 (5.85)	107 (4.39)	0.73	0.60-0.90	0.003	
	Gundog	135657 (17.04)	246 (10.08)	0.58	0.50-0.67	<.001	
	Unrecorded	5111 (0.64)	4 (0.16)	0.25	0.09-0.67	0.006	
Skull shape	Mesocephalic	385944 (48.49)	966 (39.59)				<.001
	Brachycephalic	145101 (18.23)	619 (25.37)	1.70	1.54-1.89	<.001	
	Dolichocephalic	71429 (8.97)	237 (9.71)	1.33	1.15-1.53	<.001	
	Unrecorded	193417 (24.30)	618 (25.33)	1.28	1.15-1.41	<.001	
Coat length	Short	284391 (35.73)	915 (37.50)				<.001
	Long	65717 (8.26)	187 (7.66)	0.88	0.76-1.04	0.126	

	Medium	171385 (21.53)	365 (14.96)	0.66	0.59-0.75	<.001	
	Hairless	276 (0.03)	0 (0.00)	0	0.00%	0.995	
	Unrecorded	274122 (34.44)	973 (39.88)	1.10	1.01-1.21	0.033	
Chondystrophy	Not chondystrophic	265373 (33.34)	653 (26.76)				<.001
	Chondystrophic	285342 (35.85)	1016 (41.64)	1.48	1.31-1.60	<.001	
	Unrecorded	245176 (30.81)	771 (31.60)	1.28	1.15-1.42	<.001	[1]

[1] Column percentages are shown in brackets.
*CI confidence interval
Total of 2440 cases and 795,891 non case
